# Diverse Roles of the LINC Complex in Cellular Function and Disease in the Nervous System

**DOI:** 10.3390/ijms252111525

**Published:** 2024-10-26

**Authors:** Ken-ichiro Kuwako, Sadafumi Suzuki

**Affiliations:** Department of Neural and Muscular Physiology, School of Medicine, Shimane University, 89-1 Enya-cho, Izumo-shi 693-8501, Shimane, Japan

**Keywords:** LINC complex, nuclear envelope, nervous system, diseases, cytoskeleton

## Abstract

The linker of nucleoskeleton and cytoskeleton (LINC) complex, which spans the nuclear envelope, physically connects nuclear components to the cytoskeleton and plays a pivotal role in various cellular processes, including nuclear positioning, cell migration, and chromosomal configuration. Studies have revealed that the LINC complex is essential for different aspects of the nervous system, particularly during development. The significance of the LINC complex in neural lineage cells is further corroborated by the fact that mutations in genes associated with the LINC complex have been implicated in several neurological diseases, including neurodegenerative and psychiatric disorders. In this review, we aimed to summarize the expanding knowledge of LINC complex-related neuronal functions and associated neurological diseases.

## 1. Introduction

A major difference between eukaryotes and prokaryotes is that, in eukaryotes, most genetic material is confined to the nucleus, which is separated from the cytoplasm by a double lipid bilayer nuclear envelope (NE). In addition to its central role in gene expression, the nucleus is highly involved in subcellular organization, such as organelle distribution and cell polarization, by linking with the cytoskeletal network [1]. Conversely, mechanical forces generated by the cytoskeleton are transmitted to the nucleus, influencing its structure, position, and gene expression through chromatin arrangement [2,3]. This bidirectional communication between the NE and cytoplasm orchestrates various cellular events. The mechanical connection between the nucleus and the cytoskeleton, such as actin filaments and microtubules, is established by the linker of nucleoskeleton and cytoskeleton (LINC) complex, an evolutionarily conserved molecular bridge across the NE [4,5,6].

During brain development, highly ordered cell migration accompanied by structural changes in the nucleus are crucial for establishing an intricate brain architecture [7,8]. Furthermore, neurons are highly polarized cells with precisely oriented axons and dendrites, closely linked to the intracellular positioning of the nucleus [9,10]. Therefore, the precise regulation of nuclear dynamics in neural progenitor cells, neurons, and possibly, glial cells is critical for proper brain function. In the nervous system, the major constituents of the LINC complex are highly expressed and play key roles in nuclear positioning and cell migration across various species [11,12,13,14,15,16,17,18]. Numerous studies revealed that the dysregulation of LINC complex-mediated nuclear dynamics can severely impair neural function [13,19,20,21]. Notably, beyond its well-established roles in nuclear migration and positioning, recent research highlights the LINC complex’s essential role in cell differentiation and molecular trafficking in the nervous system [22,23,24]. Furthermore, unexpected findings in *Drosophila* and *Caenorhabditis elegans* (*C. elegans*) reveal that the LINC complex is involved in synaptic transmission and axonal polarity in a nucleus-independent manner [25,26].

In humans, numerous mutations in the LINC complex gene spectrin repeat containing nuclear envelope protein 1 (*SYNE1*) have been identified in cerebellar ataxia. Furthermore, this gene mutation has been found in individuals with bipolar disorder (BD), autism spectrum disorder (ASD), and amyotrophic lateral sclerosis (ALS), underscoring the importance of the LINC complex in the nervous system [27,28,29,30]. The LINC complex may act as an effector in the pathogenesis of various genetic neurological disorders where mutations in LINC complex genes have not been reported [31,32].

Therefore, in this review, we aimed to summarize the expanding knowledge regarding the role of the LINC complex in the nervous system and related neurological diseases. We also highlighted some issues that required further elucidation in the Concluding Remarks. For an in-depth discussion on the molecular structure and classification of the LINC complex as well as the molecular basis for the LINC complex-mediated mechanotransduction and nuclear migration, readers are encouraged to consult the excellent reviews dedicated to each topic [6,15,33,34,35].

## 2. The LINC Complex

**Figure 1:**
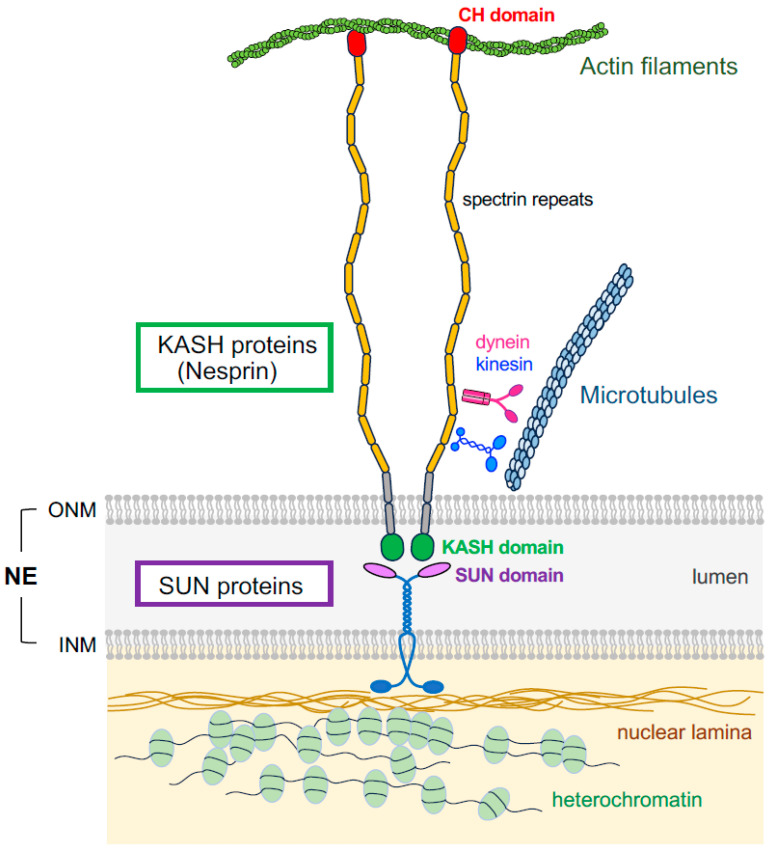
The LINC complex connecting the cytoskeleton and nuclear component. The LINC complex comprises SUN proteins in the inner nuclear membrane (INM) and KASH proteins (Nesprin in mammals) in the outer nuclear membrane (ONM). The interaction between the SUN domain of SUN proteins and the KASH domain of KASH proteins in the lumen of the nuclear envelope (NE) forms a LINC complex that physically connects the cytoskeleton and nucleoskeleton. The nucleoplasmic domain of SUN proteins is associated with the nuclear lamina, anchoring chromatin beneath the INM. On the cytoplasmic side, ONM-penetrating KASH proteins possess a long spectrin repeat structure and interact with actin filaments through the calponin homology (CH) domain and with microtubules via motor proteins, such as dynein and kinesin.

The LINC complex comprises two nuclear protein families: Sad1/UNC84 (SUN) domain-containing proteins, located on the inner nuclear membrane, and Klarsicht/Abnormal nuclear anchorage-1 (ANC-1)/SYNE homology (KASH) domain-containing proteins, located on the outer nuclear membrane (Figure 1) [36,37]. The N-terminal of SUN proteins interact with the nuclear lamina, anchoring chromatin beneath the inner nuclear membrane. In contrast, the C-terminal coiled-coil domain and the conserved SUN domain extend into the NE lumen. The C-terminal KASH domain of KASH proteins also protrudes into the NE lumen from the outer nuclear membrane, where it binds to the SUN domain of SUN proteins, forming a molecular bridge that connects the nucleoplasm and cytoplasm [34]. The large cytosolic domains of Nesprins interface with cytoskeletal elements, including actin filaments, microtubules, and intermediate filaments [5].

In mammals, five SUN proteins (SUN1–SUN5) are encoded by the *SUN1*–*5* genes. SUN1 and SUN2 are expressed ubiquitously, while SUN3, SUN4, and SUN5 exhibit testis-specific expression [6,38,39,40,41,42]. Furthermore, mammalian cells possess six KASH proteins, including nuclear envelope spectrin repeat proteins (Nesprin-1 to -4, encoded by *SYNE1*–*4*), KASH5, and the lymphocyte-restricted membrane protein [6,36]. Nesprin-1, -2, and -3 are widely expressed across various cell types in mammalian tissues; whereas, other KASH proteins show more cell-type specificity, such as epithelial and germ cells [6]. The major KASH proteins harbor a luminal C-terminal KASH domain; however, alternative splicing and multiple transcription initiation sites in *SYNE1* and *SYNE2* (encoding Nesprin-1 and Nesprin-2) generate a wide variety of isoforms that lack portions of the cytosolic region or even the KASH domain [36].

The largest isoforms of Nesprin-1 and Nesprin-2, Nesprin-1 giant and Nesprin-2 giant, are ~800–1000 kDa proteins containing a calponin homology (CH) domain at the N-terminus, which directly interacts with actin filaments, followed by numerous spectrin repeats in the cytoplasmic region (Figure 1) [43,44]. Furthermore, Nesprin-1 and -2 also anchor microtubules to the NE via direct interactions with the anterograde and retrograde motor proteins, kinesin and dynein, respectively (Figure 1) [36].

The LINC complex plays a pivotal role in diverse cellular functions through interactions with the cytoskeleton, such as the control of nuclear structural integrity, nuclear migration, and chromatin configuration. The cytoskeleton is a crucial determinant of cellular mechanical properties by mediating the physical response of the cell to surrounding cues. It also serves as a rail for the subcellular trafficking of proteins and organelles. The functions of the LINC complex are primarily mediated through the interaction of the cytoskeleton with Nesprin and SUN on the cytoplasmic and nucleoplasmic sides, respectively. These transmit mechanical forces to the nucleus and regulate nuclear motility via cytoskeleton-associated motor proteins (Figure 1).

**Figure 2:**
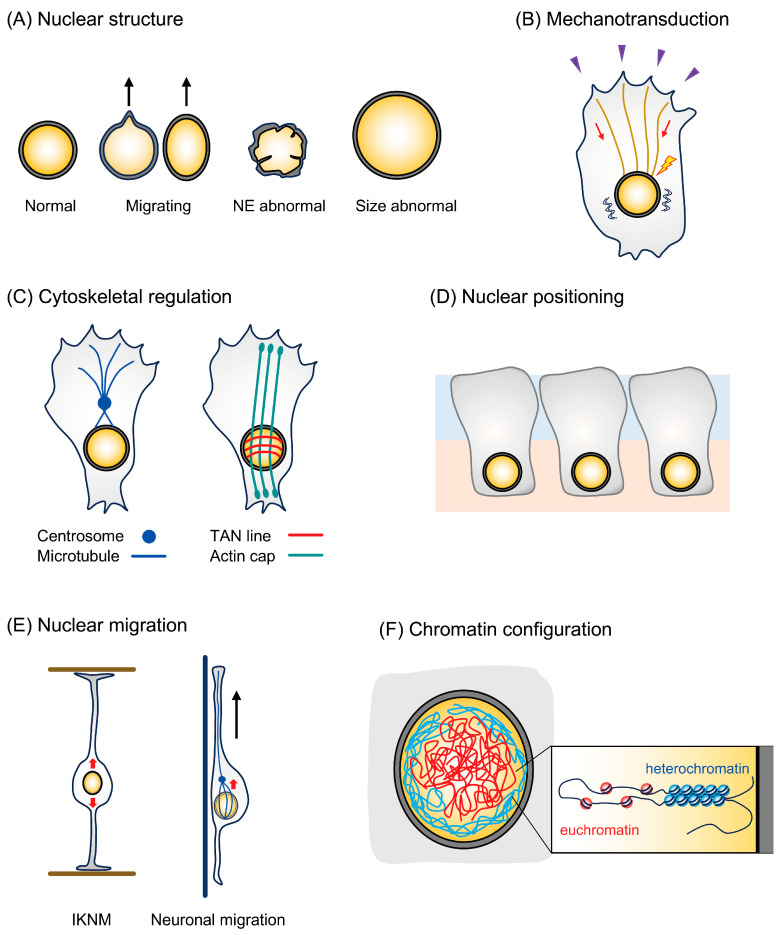
Diverse cellular functions of the LINC complex. (**A**) Nuclear structure. The LINC complex is essential for the maintenance of nuclear shape and size and for nuclear dynamics during migration, and dysfunction of the LINC complex leads to severe abnormalities in nuclear size and nuclear envelope (NE) structure. (**B**) Mechanotransduction. The LINC complex transmits mechanical forces from the external environment to the nucleus through the cytoskeletal network. (**C**) Cytoskeletal regulation. The LINC complex tethers the centrosome to the nuclear envelope via microtubules to establish the centrosome–nucleus coupling, which is important for nuclear dynamics. The LINC complex also controls the formation of perinuclear actin stress fibers involved in mechanotransduction, such as actin caps and TAN line. (**D**) Nuclear positioning. In particular cells, the LINC complex determines and maintains specific nuclear positioning via motor proteins that move on the cytoskeleton. (**E**) Nuclear migration. Proper nuclear migration during the interkinetic nuclear migration (IKNM) and neuronal migration is essential for the construction of elaborative brain structures. The LINC complex and motor proteins cooperate to move the nucleus. The red and black arrows indicate the direction of nuclear migration and cell migration, respectively. (**F**) Chromatin configuration. Chromatin is spatially arranged in a nonrandom fashion, with heterochromatin at the nuclear periphery and euchromatin farther from the nuclear envelope. Since the LINC complex tethers chromatin to the nuclear envelope via nuclear lamina, defects in the LINC complex have a major impact on chromatin configuration and, thus, global gene expression.

Since the nucleus exists under continuous tension through the cytoskeleton, any disruption of these mechanical forces on the nucleus due to the loss of the LINC complex significantly alters the nuclear structure (Figure 2A) [45]. Furthermore, the LINC complex is essential for maintaining the bilayer structure of the NE, directly influencing the overall shape and size of the nucleus. In particular, the coupling between SUN and Nesprin in the NE lumen determines the appropriate distance between the inner and outer nuclear membranes, and its defects induce NE deflection [40,46]. The LINC complex not only mediates the mechanical forces exerted by the cytoskeleton onto the nucleus but also organizes the cytoskeletal network itself (Figure 2B,C). For example, it is involved in forming perinuclear actin stress fibers, called the actin cap and the TAN line, which are essential for nuclear motility and mechanotransduction to the nucleus [33]. Furthermore, the LINC complex plays a crucial role in nuclear migration and ciliogenesis by linking the nucleus to the centrosome (also known as the microtubule organizing center) [13,47,48]. In dynamic cellular environments, nuclear motility contributes to proper cellular function. The coordination of the LINC complex with cytoskeleton motor proteins is particularly important for nuclear positioning and cell migration, where microtubule motors or actin motors control nuclear motility by pulling the nucleus directly or indirectly in specific directions within the cell (Figure 2D,E) [49,50,51]. Furthermore, since SUN proteins in the LINC complex interact with chromatin via the nuclear lamina, the complex is also involved in chromatin dynamics (Figure 2F). During meiosis, the LINC complex anchors chromatin to the NE to regulate chromosome segregation across species [52]. Moreover, the LINC complex influences gene expression via chromatin arrangement. Chromatin has a nonrandom spatial configuration in the nucleus, with transcriptionally inactive heterochromatin accumulating at the nuclear periphery and transcriptionally active euchromatin located away from the NE. Changes in the chromatin arrangement can significantly affect gene expression [53,54]. By transmitting extracellular mechanical forces to the nucleus, the LINC complex induces chromatin structure rearrangements that drive global changes in gene expression, triggering cell differentiation [22,45].

## 3. Cellular Functions of the LINC Complex in the Nervous System

### 3.1. Nuclear Positioning

Precise nuclear positioning is crucial for cell function in the nervous system. In many cell types, microtubule motor proteins translocate the nucleus in specific directions through association with the NE-tethered LINC complex, ensuring the proper intracellular positioning of the nucleus [15,51].

**Figure 3:**
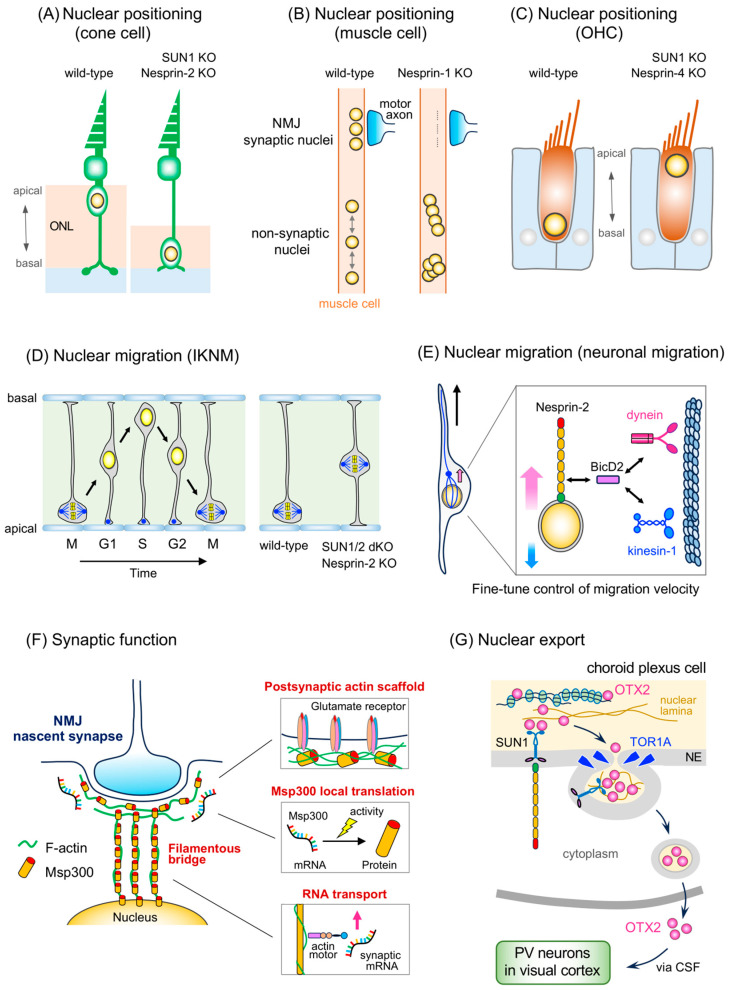
The LINC complex-mediated regulation in the nervous system. (**A**) Nuclear positioning in cone photoreceptor cell. In wild-type, the cone nuclei are located at the most apical part of the outer nuclear layer (ONL). SUN1 or Nesprin-2 knockout (KO) results in abnormal basal positioning of the cone nuclei, thereby reducing the ONL thickness. (**B**) Nuclear positioning in muscle cell. The cluster of synaptic nuclei beneath the neuromuscular junction (NMJ) is abolished in Nesprin-1-deficient muscle cells. In addition, loss of Nesprin-1 results in clusters or arrays of nonsynaptic nuclei that are uniformly distributed in wild-type. (**C**) Nuclear positioning in outer hair cell (OHC). The OHC nuclei in wild-type are located on the most basal side; whereas, Nesprin-4 or SUN1 knockout OHC nuclei are abnormally located on the apical side. (**D**) Nuclear migration. Interkinetic nuclear migration (IKNM) is a cell-cycle-dependent dynamic nuclear migration between the apical and basal surfaces of neural progenitor cells in the neuroepithelium. In SUN1/2 double knockout (dKO) or Nesprin-2 knockout mice, the nuclei are not located on the apical surface in M-phase due to IKNM abnormalities. (**E**) Nuclear migration during neuronal migration. In newly born neurons, the motor protein adaptor BicD2 mediates the interaction of Nesprin-2 to either dynein or kinesin-1. Dynein moves the nucleus toward the centrosome side, the direction of cell advance (the pink arrows), while kinesin-1 pulls the nucleus in the opposite direction to dynein (blue arrow). The cooperative action of dynein and kinesin-1 may fine-tune the velocity of nuclear migration. The centrosome is depicted as a small blue circle in the schematic diagram of a neuron. (**F**) Synaptic function. Msp300, a *Drosophila* orthologue of Nesprin-1, contributes to synaptic maturation at the NMJ through multiple mechanisms. Msp300 with F-actin forms a long filamentous bridge between the nucleus and the nascent postsynaptic site at the NMJ. Through this “bridge”, Msp300 is involved in the transport of synaptic mRNAs to the postsynaptic sites, where those mRNAs undergo local translation. Msp300 itself is also locally translated in a neural activity-dependent manner at the postsynaptic site to form a postsynaptic actin scaffold network that is required for glutamate receptor anchoring. (**G**) Nuclear export. In choroid plexus cells, SUN1 captures OTX2 transcription factor at the nuclear periphery to initiate nuclear export of OTX2. Then, OTX2 is transported to the cytoplasm through the scission of nuclear budding vesicles mediated by TOR1A, which is localized in the nuclear envelope (NE) lumen followed by extracellular secretion. OTX2 secreted from choroid plexus cells reaches parvalbumin (PV) neurons in the developing visual cortex via cerebrospinal fluid (CSF) and plays an essential role in establishing visual function.

In retinal photoreceptor cells, polarized nuclear positioning is conserved across species and is crucial for visual function. In *Drosophila*, SUN and KASH domain-containing proteins Klaroid and Klarsicht, respectively, are essential to the apical localization of photoreceptor cell nuclei in the larval eye disc. Both *Klaroid* and *Klarsicht* mutant flies exhibit very similar eye dysplasia, known as rough eyes, with abnormal nuclear positioning in photoreceptor cells (Table 1) [11,55]. Klarsicht is involved in the polarized nuclear localization in photoreceptor cells by linking the nucleus to the centrosome, likely through interactions with dynein, thereby moving the nucleus to the apical side [11,56]. Similarly, the knockdown of SYNE2a in zebrafish results in a basal displacement of photoreceptor nuclei, which is a phenocopy of the functional dynein complex inhibition (Table 1) [12]. In mice, the LINC complex is required for the apical positioning of cone photoreceptor nuclei, which reside in the outermost part of the outer nuclear layer (ONL) in the retina (Figure 3A). Deletion of Nesprin-2 or SUN1 disrupts nuclear migration toward the apical side in cone cells, leading to a significant reduction in ONL thickness (Table 1) [57]. Nesprin-2 interacts with both the dynein/dynactin complex and kinesin in the mouse retina [57], suggesting that motor protein-mediated nuclear translocation is essential for retinal development. Another study using a dominant-negative approach that strongly inhibits LINC complex function also demonstrated the overt mislocalization of cone nuclei within the ONL [58]. Further studies have revealed that the mislocalization of cone nuclei to the basal side of the ONL, resulting from LINC complex dysfunction, reduces the synaptic transmission efficiency between cone cells and bipolar cells, impairing dark adaptation. This underscores the critical role of LINC complex-mediated nuclear positioning in photoreceptor cells for visual function [20,57]. 

The LINC complex is also important for the organized anchoring of both synaptic and nonsynaptic nuclei in skeletal muscle cells, which is necessary for the proper axonal innervation of motor neurons (Figure 3B). The cluster formation of synaptic nuclei beneath the neuromuscular junction (NMJ), proposed to be essential for maintaining the postsynaptic components of the NMJ, is completely inhibited in Nesprin-1 knockout mice (Table 1) [19]. Furthermore, nonsynaptic nuclei, which are normally evenly distributed, aberrantly form clusters and arrays in Nesprin-1-deficient muscle cells [19]. Nesprin-1/-2 double knockout mice die at birth of respiratory failure exhibiting deficits in phrenic nerve innervation, which may result from the absence of synaptic nuclei in skeletal muscle cells [19].

In the cochlea, outer hair cells (OHCs) fine-tune the deflection of the basement membrane by altering their cell length, thereby enhancing the sensitivity and frequency resolution of the auditory system [59]. These refined sound wave signals are eventually converted into neuronal signals by inner hair cells and transmitted to the brain via afferent cochlear nerve fibers. In wild-type mice, OHC nuclei are strictly located at the cell basement; whereas, the nuclei of Nesprin-4- or SUN1-deficient OHCs are abnormally localized at the apical surface (Figure 3C) [60]. Nesprin-4 or SUN1 deficiency induces the mislocalization of OHC nuclei, ultimately leading to OHC degeneration during postnatal development, resulting in hearing loss (Table 1) [21,60]. Nesprin-4 is a small KASH protein with a short cytoplasmic region, lacking the CH domain (which interacts with actin filaments); however, it possesses a conserved kinesin-1 light–chain interaction domain in the cytoplasmic region and a C-terminal KASH domain essential for binding to SUN proteins [36,61]. A recent study demonstrated that kinesin-1 binds to Nesprin-4 and plays an essential role in Nesprin-4-mediated nuclear positioning in OHCs, suggesting a mechanism whereby the microtubule motor actively pulls the OHC nuclei toward the cell basement via the LINC complex [61].

In *C. elegans*, the position of neuronal cell bodies within tissues is maintained by a mechanism that protects neurons from excessive extracellular forces, such as muscle contraction. ANC-1, a KASH protein in *C. elegans*, is involved in nuclear anchoring, as in mammalian cells [6]. Notably, in *ANC-1* mutant worms, lumbar neurons are misaligned, implying that proper nuclear anchoring may make the neuronal cell body less susceptible to mechanical forces that deleteriously displace the cell body (Table 1) [62].

### 3.2. Nuclear and Cell Migration

Interkinetic nuclear migration (IKNM) refers to the dynamic, cell-cycle-dependent nuclear oscillation between the apical and basal surfaces of neural progenitor cells within the neuroepithelium [63,64]. For example, in radial glial progenitor cells of the developing cerebral cortex, M-phase nuclei are located at the apical (ventricular) surface; whereas, nuclei in the S-phase are located at the basal (pial) surface. Nuclei in the G1 and G2 phases are positioned at the intermediate area (Figure 3D) [15,64]. During IKNM, the centrosome remains anchored at the apical surface, aligning microtubules with their plus end oriented toward the basal surface. Microtubule motors, such as dynein and kinesin, along with the actin motor myosin II, play pivotal roles in nuclear oscillations along the apical–basal axis [64].

During photoreceptor cell differentiation in the retina, the nuclei of retinal progenitor cells translocate between the basal and apical surfaces of the neuroblastic layer in synchrony with the cell cycle [63]. In Nesprin-2 knockout mice and SUN1/2 double knockout mice, this IKNM in retinal progenitor cells is disrupted, as the nuclei of M-phase mitotic cells are sparsely located within the neuroblastic layer (Table 1) [57]. Since Nesprin-2 interacts with dynein and kinesin in the developing retina, microtubule motors are likely to mediate upward and downward nuclear movements in retinal progenitor cells [57].

The loss of Nesprin-2 or SUN1/2 is also detrimental to IKNM in radial glial progenitors of the developing cerebral cortex in mice (Figure 3D) (Table 1) [13]. In Nesprin-2 knockout or SUN1/2 double knockout mice, a significant number of mitotic progenitor cells are mislocalized in the ventricular zone, leading to the progressive depletion of neural progenitor cells [13]. Furthermore, newly born neurons fail to migrate normally along radial glial fibers in these mice (Table 1). Consequently, SUN1/2- or Nesprin-1/2-deficient brains display severe abnormalities, including enlarged lateral ventricles, inverted layers, and reduced brain size [13]. Another study in zebrafish demonstrated that SUN1 knockdown disrupted the basal-to-apical nuclear migration of neural progenitor cells in the ventricular zone of the developing brain, indicating that the LINC complex is involved in IKNM across species (Table 1) [17].

As observed in the developing retina [57], Nesprin-2 interacts with dynein and kinesin in the embryonic brain, suggesting that microtubule motors play a central role in both the IKNM of progenitor cells and the nucleokinesis of migrating neurons in the developing cerebral cortex [13]. Recent studies in mice and rats have further elucidated the molecular mechanisms of the Nesprin-2-mediated IKNM and neuronal migration in the cerebral cortex. Nesprin-2 recruits dynein to the NE through its interaction with the motor protein adaptor Bicaudal D2 (BicD2), facilitating both the IKNM of radial glial progenitors and the migration of newly born neurons (Figure 3E) [18,32,65]. RNA-binding protein 2 (RanBP2), a nucleoporin, also binds to BicD2 in competition with Nesprin-2 and recruits dynein to the nuclear pore. Thus, maintaining a proper balance between BicD2 binding to Nesprin-2 and to RanBP2 is important for regulating both nuclear and neuronal migration [32]. Interestingly, unlike in fibroblasts, actin binding is dispensable for Nesprin-2-mediated neuronal migration [18,66]. Moreover, the mechanical coupling between the centrosome and nucleus is disrupted in SUN1/SUN2- or Nesprin-1/2-deficient glial cells isolated from the embryonic brain [13]; however, the centrosome’s advance toward the basal surface in migrating neurons occurs independently of Nesprin-2-mediated nuclear movement [18]. In addition to dynein, kinesin-1 also binds to Nesprin-2 either directly or via BicD2; however, its action exerts an opposing effect, retarding nuclear migration toward the basal surface in migrating neurons [18]. These findings suggest that the Nesprin-2-BicD2 system fine-tunes the velocity of nuclear migration through the coordinated actions of dynein and kinesin-1 independently of centrosome advance [18].

A detailed analysis of nuclear movement in migrating neurons has revealed a novel dynamic motion of the nucleus mediated by the LINC complex [16]. In migrating cerebellar granule neurons, the nucleus rotates in the direction of its translocation toward the leading process, independently of centrosome motility. The spin torque for rotation is produced by dynein and kinesin-1 and is transmitted to the nucleus via the LINC complex, independently of actomyosin. The LINC complex is required for both nuclear rotation and translocation in granule neurons, as the functional inhibition of Nesprins using a dominant-negative tool impedes both types of nuclear movement (Table 1) [16]. Kinesin-1 appears to exert unbalanced point forces on the NE, pulling the nucleus via its interactions with Nesprins. As inferred from its importance in nuclear dynamic control, the loss of kinesin-1 impairs granule cell migration in the developing cerebellum. In addition, similar to its function in newly born migrating cortical neurons [18], the Nesprin-2-BicD2 system plays a crucial role in continuous nuclear movement along advancing microtubules in migrating cerebellar granule neurons by coordinating the interplay of kinesin-1 and dynein [67]. Thus, these studies also highlight the essential roles of the LINC complex-mediated recruitment of microtubule motors to the NE in nuclear motility control [16,67,68].

### 3.3. Synaptic Functions

Two Nesprin-1 orthologues, the ANC-1 in *C. elegans* and muscle-specific protein 300 (Msp300) in *Drosophila*, are involved in synaptic functions. Similar to the Nesprin-1 giant, both ANC-1 and Msp300 are large NE-tethered proteins characterized by a C-terminal KASH domain and an N-terminal actin-binding domain [4].

ANC-1 is involved in the synapse formation of the GABAergic dorsal D motor neurons and also the axon termination of the posterior lateral microtubule mechanosensory neurons in *C. elegans* (Table 1) [69]. Through proteomic and genetic approaches, a molecular pathway for ANC-1-mediated synaptic and axonal functions has been elucidated. ANC-1, in conjunction with the signaling molecule RPM1 at the NE, regulates the nuclear import of the canonical β-catenin BAR-1 and modulates its activity, which may influence gene expression via the transcription factor POP-1 [69]. However, the detailed mechanisms of ANC-1-mediated BAR-1 nuclear import and the subsequent events leading to synapse formation and axon termination are still unknown.

Msp300 is crucial for several aspects of synaptic function at the NMJ (Figure 3F) (Table 1). Msp300 is anchored to the NE of muscle cells via its KASH domain and is essential for myonuclear anchoring at the NMJ [25]. Notably, Msp300 forms an extensive filamentous network containing F-actin, which spans from the nucleus to the postsynaptic elements at the NMJ [70].

These filaments emanate from the nucleus, specifically wrapping around nascent boutons at the synaptic site, forming a postsynaptic actin scaffold that is required to control the synaptic density of glutamate receptors at the larval NMJ [70,71]. In *Msp300* mutant larvae, the amount of GluRIIA, a glutamate receptor, is significantly reduced at the NMJ postsynapses, leading to impaired neurotransmission and locomotor defects, underscoring the importance of the Msp300-mediated organization of a postsynaptic actin network for synaptic function [25]. Furthermore, Msp300 is involved in the transport of synaptic messenger RNAs (mRNAs), which are locally translated at postsynapses through filamentous bridges between the nucleus and the synapse. Msp300 binds to synaptic RNAs and regulates the appropriate levels of postsynaptic mRNAs in coordination with the unconventional actin motor myosin 31DF [70]. The Msp300-dependent mRNA transport to postsynaptic sites is crucial for synaptic bouton maturation and activity-dependent plasticity at the NMJ [70]. Notably, a recent study revealed that Msp300 itself is locally translated at synaptic sites in the NMJ in a neural activity-dependent manner [72]. Msp300 proteins are relatively scarce at mature NMJ synapses but are highly enriched at nascent synapses in response to elevated neural activity [70]. Msp300 mRNAs, along with the RNA-binding protein Syncrip and translation factors such as eIF4E, are localized near nascent synapses and are locally translated in an activity-dependent manner [72]. This mechanism allows for the rapid mobilization of the Msp300 protein to nascent synaptic boutons, helping to organize an actin scaffold for postsynaptic maturation. Collectively, Msp300 is significantly involved in NMJ synaptic function through multiple mechanisms.

In mice, a 102-kDa brain-specific splicing variant of Nesprin-1 that is not anchored to the NE, termed candidate plasticity gene 2 (CPG2), has been identified as crucial for synaptic function (Table 1). CPG2 is the spectrin-repeat-only Nesprin-1 variant lacking the KASH domain and was originally characterized as an activity-dependent transcript [73]. The expression of CPG2 in the brain is induced by light stimulation and kainate administration, which activate glutamate receptors [73,74]. Furthermore, CPG2 is upregulated in the cerebral cortex during postnatal development [74]. Subsequent studies have revealed that CPG2 is localized to the postsynaptic endocytotic zone of excitatory synapses and is involved in postsynaptic endocytosis [75,76]. The downregulation of CPG2 hampers the internalization of the α-amino-3-hydroxy-5-methyl-4-isoxazoleproprionic acid receptor (AMPAR), a glutamate receptor, and causes a reduction in dendritic spine head size [75]. Despite lacking the conserved CH domain responsible for binding actin filaments, CPG2 is able to associate with F-actin through novel bipartite coiled-coil domains located at its C-terminus [76]. Phosphorylation of the actin-binding domain of CPG2 by protein kinase A crucially regulates the CPG2-mediated internalization of AMPARs and postsynaptic strength [76].

Thus, given the essential synaptic functions of the non-nuclear Nesprin-1 isoform CPG2 and the diverse splicing mechanisms of mammalian Nesprin-1/-2, other important Nesprin isoforms with neural functions remain to be identified.

### 3.4. Other Neural Functions

In addition to its role in regulating nuclear motility and synaptic function, the LINC complex is involved in various neuronal processes, including neural/glial differentiation, polarized axon growth, and nuclear export.

Oligodendrocyte progenitor cells (OPCs) undergo differentiation upon mechanical stimulation that induces global chromatin remodeling and subsequent gene expression changes [77]. During OPC differentiation in mice, the expression of SUN1 and Nesprin-1 increases [22], with mechanical stimulation further upregulating SUN1 levels in OPCs [77]. The knockdown of Nesprin-1 in OPCs leads to impaired heterochromatin formation and glial differentiation, suggesting that the LINC complex regulates oligodendrocyte maturation by transducing external mechanical forces to the nucleus, leading to alterations in the epigenetic landscape (Table 1) [22].

The membrane diffusion barrier of the endoplasmic reticulum (ER) controls the segregation of aging factors, such as damaged proteins, in proliferative neural stem cells (NSCs) within the hippocampus, and the barrier strength in NSCs diminishes with age [78]. In aged hippocampal NSCs, SUN1 expression is increased compared with that in young NSCs, with a concomitant decrease in the nuclear lamina protein lamin B1 [79]. Restoring the balance between SUN1 and lamin B1 expression in aged NSCs, either by SUN1 knockdown or lamin B1 overexpression, can improve ER barrier strength, leading to enhanced NSC proliferation and neurogenesis in the aged hippocampus (Table 1) [79]. The mechanism through which NE-related molecules such as the LINC complex and lamin regulate ER barrier strength remains to be fully elucidated; however, this system contributes significantly to the differentiation potential of NSCs, and its disruption with age may contribute to declines in brain function.

In *C. elegans*, the protein ANC-1 regulates the polarized axon growth of touch receptor ALM neurons by facilitating the recruitment of mitochondria to the base of the proximal axon (Table 1) [26]. The role of ANC-1 is mediated by two recently identified isoforms, ANC-1A and ANC-1C, which both contain an N-terminal CH domain that interacts with actin filaments. Despite sharing most domains other than the N-terminal with ANC-1B, which is known for its role in nuclear anchoring, ANC-1A and ANC-1C are reported to localize to the cytoplasm; however, whether these two isoforms are associated with the nucleus and how they recruit mitochondria to the base of the axon remains unclear [26].

During the development of visual function in mice, the intercellular transfer of the orthodenticle homeobox 2 (OTX2) transcription factor to the developing visual cortex is crucial for the maturation of inhibitory cortical circuits and the initiation of the critical period for plasticity [80]. OTX2 is secreted by choroid plexus cells and transferred to parvalbumin-expressing neurons in the visual cortex [24,81]. Recent research has demonstrated that OTX2 binds to SUN1 within the nucleus [24]. Torsin-1a (TOR1A), an AAA+ ATPase located in the lumen of the NE, regulates the scission of nuclear budding vesicles. TOR1A captures LINC complex-associated OTX2 and facilitates the subsequent nuclear-to-lysosomal transport of OTX2 for degradation and further secretion into the extracellular space (Figure 3G). The inhibition of the LINC complex function through the forced expression of a dominant-negative KASH protein suppresses OTX2 secretion, highlighting the importance of OTX2 binding to the LINC complex for TOR1A-mediated transport (Table 1) [24]. Furthermore, inhibiting the LINC complex results in a reduction in parvalbumin neurons within the visual cortex and impairs visual acuity, underscoring the necessity of OTX2 nuclear export via the LINC complex, followed by extracellular secretion, which is indispensable for establishing visual function [24].

## 4. The LINC Complex-Related Neurological Diseases

### 4.1. The LINC Complex Mutation-Associated Diseases

Mutations in LINC complex genes are implicated in several neurological and muscular degenerative diseases, including Emery–Dreifuss muscular dystrophy (EDMD) and dilated cardiomyopathy (DCM) (Table 2) [82]. Notably, in LINC complex-related diseases, most mutations are confined to the *SYNE1* gene. Over 90% of disease-associated *SYNE1* mutations occur in the coding region, with approximately half being nonsense mutations and about one-fourth being frameshift mutations [30]. Intronic *SYNE1* mutations can affect gene splicing, potentially leading to the premature termination of Nesprin-1 protein synthesis. The molecular etiology of each disease caused by *SYNE1* mutations has not been elucidated; however, loss-of-function due to the absence of Nesprin-1 expression or the production of truncated, nonfunctional Nesprin-1 proteins is thought to be the underlying pathogenic mechanism. 

The most prevalent disease resulting from *SYNE1* mutations is autosomal recessive cerebellar ataxia type 8 (SCAR8), also known as autosomal recessive cerebellar ataxia type 1 or recessive ataxia of Beauce. Recent studies indicate that over 85% of disease-associated *SYNE1* mutations have been identified in SCAR8, surpassing the mutation frequency found in EDMD and DCM [30]. SCAR8 was initially discovered in 26 French Canadian families, comprising 53 affected individuals who exhibited late-onset and slow-progressing relatively pure cerebellar ataxia [83]. SCAR8 cases have since been reported globally, predominantly in European, Middle Eastern, and East Asian countries [84,85,86,87,88,89,90,91]. Disease-associated *SYNE1* mutations in SCAR8 are distributed throughout the gene, including the CH domain, but notably absent from the KASH domain [87].

As noted above, the mechanism by which *SYNE1* mutations cause SCAR8 remains unclear; however, one possibility involves a specific isoform of Nesprin-1 that is highly enriched in the cerebellum. Razafsky et al. identified a Nesprin-1 giant isoform, termed KLNes1g, which lacks the KASH domain [92]. KLNes1g is most abundantly expressed in the cerebellum and localizes not to the NE but to synapses between mossy fibers and cerebellar granule neurons, which are essential for cerebellar function. Furthermore, KLNes1g is localized to synaptic vesicles within mossy fiber axonal terminals, known as glomeruli, and binds to clathrin, which coats the synaptic vesicles. This suggests that KLNes1g may exert a synaptic function within cerebellar circuits [92]. Thus, it is possible that *SYNE1* mutations in SCAR8 significantly impact KLNes1g expression and function in the cerebellum; however, the direct involvement of KLNes1g in the pathogenesis of SCAR8 has not yet been demonstrated.

Despite numerous *SYNE1* gene mutations in SCAR8, Nesprin-1 knockout mice do not exhibit SCAR8 pathology [13]. The molecular basis for the phenotypic differences between humans and mice in the loss of Nesprin-1 function remains to be elucidated, but it is conceivable that functional complementation by Nesprin-2 may differ between species. Conversely, SUN1 knockout mice exhibit cerebellar ataxia; although, no mutations in the *SUN1* gene have been identified in SCAR8. In SUN1 knockout mice, Nesprin-2 is mislocalized from the NE in cerebellar Purkinje cells, leading to abnormalities in Purkinje cell migration, dendritic morphology, and synaptic distribution [93]. Furthermore, the additional genetic deletion of SUN2 to SUN1 deficiency exacerbates cerebellar ataxia [93]. SCAR8-associated mutations in LINC complex genes do not directly align with the phenotypes of the respective LINC complex gene knockout mice; however, the cumulative evidence strongly suggests that the LINC complex is crucial for motor function.

Comprehensive genomic analyses have identified *SYNE1* as a potential risk gene for psychiatric disorders, including BD and ASD. *SYNE1* gene mutations have also been found in several patients with sporadic ALS.

A large-scale genome-wide association study (GWAS) involving 7481 patients with BD identified primary association signals that reached genome-wide significance within the *SYNE1* gene region [27]. Subsequent GWAS analyses confirmed the *SYNE1* gene locus as one of the four strongest BD association loci in the genome [94]. Furthermore, accumulated evidence from further GWAS analyses suggest that BD-associated mutations in the *SYNE1* gene are primarily localized at the region encompassing a brain-specific transcript encoding CPG2 protein, which localizes to excitatory postsynapses and regulates AMPAR endocytosis as previously mentioned [28,75,76,95,96]. Many BD-associated *SYNE1* mutations have been identified in both the coding and promoter regions of the CPG2 locus, and mutations in the promoter region negatively regulate CPG2 transcription [28,96], while several mutations in the coding region attenuate the synaptic localization of the CPG2 protein and the internalization of AMPAR [96]. Notably, CPG2 protein levels were significantly reduced in the postmortem brains of patients with BD [96]. This evidence strongly suggests that the loss of CPG2 function due to BD-associated mutations in the CPG2 locus in the *SYNE1* gene may underlie the molecular etiology of BD.

The exome sequencing of patients with sporadic ASD initially identified de novo missense *SYNE1* mutations, specifically affecting the CH domain of Nesprin-1 [97]. Subsequently, a familial ASD-associated biallelic *SYNE1* mutation was identified in the spectrin repeat domain of Nesprin-1 giant [98]. To date, numerous clinically relevant *SYNE1* mutations associated with ASD have been reported and distributed throughout the gene (refer to the SFARI Gene database, https://gene.sfari.org, accessed on 2 October 2024). Remarkably, biallelic heterozygous missense *SYNE2* mutations in the spectrin repeat domain of Nesprin-2 giant were recently identified in a patient clinically diagnosed with ASD, along with developmental delay and intellectual disability [99]. In the patient-derived lymphoblastoid cells, the expression of Nesprin-2 giant with ASD mutations was significantly reduced; although, its NE localization remained unchanged, suggesting that these ASD-related mutations may impair the expression of Nesprin-2, potentially contributing to ASD pathogenesis [99].

Several *SYNE1* mutations have been reported in patients with ALS exhibiting complex symptoms, such as spastic ataxic gait or cognitive decline [29,100]. Moreover, recent studies have demonstrated that in motor neurons, cortical neurons, and spinal cord organoids harboring the ALS-causative *C9ORF72* mutation, which is most frequent in familial ALS, the LINC complex proteins, including SUN1/2 and Nesprin-1/-2, mislocalize to the cytoplasm, causing morphological alterations of the nucleus [101]. Similar abnormalities also occur in the postmortem spinal cord and motor cortex from patients with sporadic and *C9ORF72* ALS [101]. Another recent study demonstrated that SUN1 may facilitate chromatin modification protein 7 (CHMP7)-mediated toxicity in sporadic ALS. In neurons from sporadic ALS-derived induced pluripotent stem cells, SUN1 promotes the nuclear influx of CHMP7 triggered by the impaired permeability barrier of the nuclear pore complex (NPC). This influx leads to NPC injury and downstream TAR DNA-binding protein 43 dysfunction [102]. Thus, the role of ALS-associated *SYNE1* mutations in the pathogenesis of ALS remains unclear; however, the LINC complex may play multiple important roles in the ALS pathogenic cascade, causing neurotoxicity.

As noted above, Nesprin-4 is essential for the basal anchoring of nuclei in OHCs, and its loss of function causes OHC cell death and subsequent hearing loss in mice [60,61]. Since phenotypes similar to Nesprin-4 deficiency have been observed in SUN1 knockout mice, in all probability, Nesprin-4 functions by tethering to the NE via SUN1 in OHCs [60]. These findings were prompted by the identification of homozygous mutations in the *SYNE4* gene, which yielded truncated Nesprin-4 proteins that failed to localize at the NE, in patients with hereditary deafness from two families of Iraqi Jewish ancestry [60]. Recently, gene therapy using adeno-associated viral vectors to induce full-length Nesprin-4 expression in a Nesprin-4 knockout mice deafness model has been shown to restore nuclear positioning in OHCs and hearing ability significantly [21]. Reports of *SYNE4* mutations causing hereditary hearing loss are limited; however, this case is one of the few LINC complex-associated diseases where the pathogenesis is relatively well understood, with both human and mouse models exhibiting well-matched pathologies.

### 4.2. Involvement of the LINC Complex in Other Genetic Diseases

In EDMD, several *SUN1* or *SUN2* mutations increase disease severity in patients carrying other EDMD-related gene mutations, such as *LMNA* (encoding lamin A/C), *EMD* (encoding emerin), and *MYBPC3* (encoding myosin-binding protein C3), suggesting that *SUN1* and *SUN2* function as disease modifier genes [103]. Furthermore, the LINC complex may act as an important effector in other genetic neurological diseases without LINC complex gene mutation [31,47,65].

Recessive TOR1A disease is characterized by severe arthrogryposis at birth, followed by intellectual disabilities. As previously mentioned, TOR1A interacts with the LINC complex to regulate the nuclear export of OTX2, a process essential for establishing visual function [24]. Beyond its role in nuclear export, TOR1A is also implicated in brain development through another mechanism involving the LINC complex. In the proliferative zone of the fetal brain in TOR1A knockout mice, a genetic model for recessive TOR1A disease, LINC complex components, including SUN1, SUN2, and Nesprin-2 giant, are abnormally upregulated in the NE, resulting in excess neurogenesis via abnormalities in radial glial progenitor cells, eventually leading to anomalous brain structure [31]. Notably, genetically reducing SUN2 expression significantly suppressed abnormal brain morphogenesis in TOR1A knockout mice, underscoring the critical role of the LINC complex in the disease’s pathogenic cascade.

Heterozygous missense mutations in the *BICD2* gene primarily cause autosomal-dominant lower-extremity-predominant spinal muscular atrophy type 2, characterized by reduced spinal motor neurons and muscle weakness, without brain malformation. Recently, a novel de novo BicD2 nonsense mutation, K775X, was identified in a patient with lissencephaly [65]. BicD2 regulates the migration of newly born neurons in the developing cortex by recruiting dynein and kinesin-1 to Nesprin-2 on the NE for nuclear translocation; however, the K775X mutation of BicD2 disrupts its interaction with Nesprin-2, severely inhibiting neuronal migration [18,32,65]. This evidence indicates that the BicD2-Nesprin-2 pathway is crucial for normal cortical development, and its abnormalities can lead to lissencephaly.

Meckel–Gruber syndrome (MKS), a severe recessive disease characterized by renal cystic dysplasia and neural developmental defects, is caused by mutations in genes that encode components of the primary cilium, classifying it as a ciliopathy. *Meckelin* is one of the MKS pathogenic genes involved, and its transcript is essential for cilia formation. Meckelin regulates the subcellular localization of Nesprin-2 via actin filament-mediated binding, and the loss of Nesprin-2 results in cilia hypoplasia, suggesting that Nesprin-2 may act as an effector in MKS pathogenesis due to Meckelin dysfunction [47].

## 5. Concluding Remarks

The LINC complex has been shown to regulate nuclear positioning, IKNM, and cell migration in various nervous system cell types, including sensory receptor cells, NMJ synapse cells, neural progenitor cells, and young neurons via nuclear motility control. Similar to its role in non-neuronal cells, LINC complex-mediated nuclear migration in the nervous system primarily involves the regulation of nuclear motility via interactions with cytoskeletal motors, particularly the microtubule motors dynein and kinesin. Furthermore, the LINC complex exhibits nervous system-specific functions, such as synaptic and axonal regulation. For example, a striking mechanism has been demonstrated in *Drosophila*, where the Msp300 contributes to synaptic maturation by forming a molecular bridge between the nucleus and synapse, regulating mRNA transport [70]. Moreover, mutations in LINC complex genes have been revealed to contribute not only to cerebellar ataxia but also to the pathogenesis of neurological diseases, such as BD and ASD. Recent reports suggest that the LINC complex may play key roles in the etiology of other genetic disorders that did not involve genetic mutations in the LINC complex, further deepening our understanding of the LINC complex’s critical role in human diseases.

Despite significant advances in understanding the neuronal functions of the LINC complex and its involvement in neurological diseases, many fundamental questions about the roles of the LINC complex in the nervous system remain unresolved. A major gap lies in the lack of comprehensive spatiotemporal expression profiles of SUN and KASH family molecules in neural lineage cells, including diverse neuron types.

Furthermore, much of the identified roles of the LINC complex so far are related to cellular events during neural development, leaving its impact on the mature nervous system largely unknown. For example, in inflammation-responsive migrating cells in the brain, such as astrocytes and microglia, the regulation of nuclear motility via the LINC complex is likely essential; however, nothing has been reported on its function in these cell types. Similarly, the physiological roles of the LINC complex in various mature neurons across different brain regions remain unclear but are of great importance to elucidate. Moreover, the molecular mechanisms through which the LINC complex contributes to the pathogenesis of neurological diseases remain largely elusive.

Recent discoveries highlighting the diverse functions of the LINC complex across species suggest that the LINC complex has many unknown functions, even in mature neurons. For example, an interesting area of research would be the possible involvement of the LINC complex in neuronal aging. Given that the LINC complex helps maintain nuclear structural integrity by adequately transmitting mechanical forces to the nucleus, age-related alterations in the LINC complex in mature neurons could significantly impact nuclear structure. Such detrimental structural changes of the nucleus with aging may, in turn, trigger global gene expression changes via the remodeling of the chromatin configuration, ultimately leading to a decline in cellular and brain function. Indeed, NE proteins are central in cellular aging, and the involvement of LINC complex molecules in cellular aging processes has been reported in non-neuronal cells [104]. For example, abnormal accumulation of SUN1 in the Golgi apparatus accelerates fibroblast aging in Hutchinson–Gilford progeria syndrome [105]. Furthermore, SUN1 has been implicated in the age-related decline in differentiation potential in adult hippocampal NSCs, as mentioned above [79].

Further studies are expected to reveal not only the detailed molecular mechanisms of the LINC complex in neural function and the pathogenesis of neurological diseases but also its involvement in lots of important processes in neural development and homeostasis.

## Figures and Tables

**Table 1:** Summary of the LINC complex function in the nervous system.

	Molecule	Classification	Function	Cell Type
**Mouse**	SUN1	SUN protein	Nuclear positioning	Photoreceptor cell Hair cell
			Nuclear migration	Radial glial progenitor Retinal progenitor
			Cell differentiation	Adult neural stem cell
			Nuclear export	Choroid plexus cell
	SUN2	SUN protein	Nuclear migration	Radial glial progenitor Retinal progenitor
	Nesprin-1	KASH protein	Nuclear positioning	Muscle cell (NMJ)
			Cell differentiation	Oligodendrocyte progenitor
	Nesprin-2	KASH protein	Nuclear positioning	Photoreceptor cell
			Nuclear migration	Radial glial progenitor Retinal progenitor Cortical neuron Cerebellar granule neuron
	Nesprin-4	KASH protein	Nuclear positioning	Hair cell
	CPG2	KASH protein (related variant)	Postsynaptic regulation	Hippocampal neuron
**Zebrafish**	SUN1	SUN protein	Nuclear migration	Neural progenitor
	SYNE2a	KASH protein	Nuclear positioning	Photoreceptor cell
***Drosophila***	Klaroid	SUN protein	Nuclear positioning	Photoreceptor cell
	Klarsicht	KASH protein	Nuclear positioning	Photoreceptor cell
	Msp300	KASH protein	Synapse formation	Muscle cell (NMJ)
***C. elegans***	ANC-1	KASH protein	Cell body positioning	Lumbar neuron
			Synapse formation	Motor neuron
			Axon termination	Mechanosensory neuron
			Axon growth	Touch receptor ALM neuron

**Table 2:** The LINC complex-associated neurological diseases.

	Neurological Disease Mutation			Non-Neurological Disease Mutation
	(1) Pathogenic Mutation	(2) Risk Mutation	(3) No Mutation (Effector?)	
***SYNE1***	SCAR8	BD, ASD, ALS	(-)	EDMD, DCM, Arthrogryposis
***SYNE2***	(-)	ASD	Lissencephaly, MKS	EDMD
***SYNE4***	Hearing loss	(-)	(-)	(-)
***SUN1***	(-)	(-)	(-)	EDMD (modifier)
***SUN2***	(-)	(-)	TOR1A disease	EDMD (modifier)

Neurological diseases involving the LINC complex are classified as (1) pathogenic mutations, (2) risk mutations, and (3) possible effectors of pathogenesis (no mutation). LINC complex genes, not listed herein, have not been reported to be involved in neurological diseases. The rightmost column shows the main disease mutations other than neurological diseases. The pathogenic mutations are highlighted in blue. SCAR8: autosomal recessive cerebellar ataxia type 8; BD: bipolar disorder; ASD: autism spectrum disorder; ALS: amyotrophic lateral sclerosis; MKS: Meckel–Gruber syndrome; EDMD: Emery–Dreifuss muscular dystrophy; DCM: dilated cardiomyopathy.
